# Chronic Infections: A Possible Scenario for Autophagy and Senescence Cross-Talk

**DOI:** 10.3390/cells7100162

**Published:** 2018-10-10

**Authors:** Milton O. Aguilera, Laura R. Delgui, Patricia S. Romano, María I. Colombo

**Affiliations:** 1 IHEM, Universidad Nacional de Cuyo, CONICET, Facultad de Ciencias Médicas, Mendoza 5500, Argentina; ldelgui@yahoo.com (L.R.D.); romanopatricia33@gmail.com (P.S.R.); mcolombo@fcm.uncu.edu.ar (M.I.C.); 2 Facultad de Odontología, Universidad Nacional de Cuyo, Mendoza 5500, Argentina; 3 Facultad de Ciencias Exactas y Naturales, Universidad Nacional de Cuyo, Mendoza 5500, Argentina; 4 Facultad de Ciencias Médicas, Universidad Nacional de Cuyo, Mendoza 5500, Argentina

**Keywords:** aging, senescence, autophagy, immune system, immunosenescence, pathogens, chronic infections

## Abstract

Multiple tissues and systems in the organism undergo modifications during aging due to an accumulation of damaged proteins, lipids, and genetic material. To counteract this process, the cells are equipped with specific mechanisms, such as autophagy and senescence. Particularly, the immune system undergoes a process called immunosenescence, giving rise to a chronic inflammatory status of the organism, with a decreased ability to counteract antigens. The obvious result of this process is a reduced defence capacity. Currently, there is evidence that some pathogens are able to accelerate the immunosenescence process for their own benefit. Although to date numerous reports show the autophagy–senescence relationship, or the connection between pathogens with autophagy or senescence, the link between the three actors remains unexplored. In this review, we have summarized current knowledge about important issues related to aging, senescence, and autophagy.

## 1. Introduction

The functionality of organs such as the brain or the immune system differs when comparing young and old individuals. Those differences are due to changes at both, molecular level (loss of chromatin, accumulation of mutations in the DNA, protein oxidation and misfolding), and at the cellular level (increased oxidative stress, changes in the membrane fluidity, aggregation of proteins, and accumulation of damaged organelles). All together, these changes generate a gradual change in the functionality of the entire organism, a process called aging.

In multicellular organisms, the aging process starts at the end of development, when tissues and organs reach the maximal and final stage of differentiation. At this point, tissues are classified into those formed by non-proliferative cells (they have a post-mitotic arrest) and those composed by proliferative cells bearing the ability of regeneration (mitotic cells). Along the lifetime, both types of tissues accumulate damage, though with different consequences. The effect of aging on mitotic cells are evidenced by an increase in the incidence of cancer after the age of 50 due to accumulation of mutations by DNA damage, while on non-mitotic cells, the main effects are the appearance of neurodegenerative disorders or cancer with increased rates after the age of 70 mainly due to the presence of damaged proteins and organelles [1].

Cells are equipped with a plethora of strategies to counteract the effects of aging by degradation and recycling of damaged organelles and molecules. Autophagy, together with the ubiquitin/proteasome system are the major degradative pathways within the cells [2]. Moreover, autophagy has the capacity to eliminate proteins and old or damaged organelles, a role named quality control function [3] by which the pathway prevents the accumulation of agents that could potentially damage DNA. Besides, there is evidence that autophagic proteins are directly involved in the maintenance of DNA stability [4,5]. This degradative process is mediated through three different mechanisms i.e., chaperone-mediated autophagy (CMA), microautophagy, and macroautophagy [2].

CMA is a selective process based on the recognition of specific KFERG-like motifs. This multistep process requires (1) substrate recognition and target to lysosomes; (2) binding and unfolding; (3) translocation and (4) degradation in the lumen of lysosomes [3,6,7]. The two key molecules involved in CMA are Hsp70 (Heat shock protein 70) and LAMP2A (Lysosomal associated membrane protein 2 A). The recognition and binding of the substrate occurs in the cytoplasm by Hsp70 and subsequently this complex binds to Lamp2A in the lysosome membrane. This complex together with other proteins participate in the transport of the previously mentioned unfolded substrate [8] to the lysosomal lumen where it is degraded [3,6,7]. 

Microautophagy refers to the process involving membrane modification in the lysosomes (protrusions or invaginations) where different substrates are trapped and finally degraded [9]. Microautophagy breaks down proteins and pieces of organelles (mitochondria, peroxisome, and lipid droplet) [10]. This mechanism has been mainly described in yeast, and in mammals, a similar mechanism has been observed, but associated with late endosomes/multivesicular bodies (LE/MVB) and termed endosomal microautophagy (eMI) [11]. eMI depends on the ESCRT machinery and surprisingly in some cases of Hsp70 (and KFERG like motif) [11]. Interestingly, the pool of substrates bound to Hsp70 that is degraded by this pathway does not depend on the unfolded state [8,11] and in mammals different from yeast, the KFERG-like motif is necessary but not sufficient to target the substrates into endosomes [12]. An extra unknown signal is necessary to the targeting of Hsp70 bound substrates to eMI. 

Macroutophagy (hereafter called autophagy) begins with the generation of a structure called isolation membrane, which is then closed generating a double membrane vesicle. In these vesicles called autophagosomes, the material to be degraded is sequestered and placed in contact with degradative enzymes after the fusion of autophagosomes with late endosomes and lysosomes [13]. During the process, several protein complexes participate and regulate different steps of the pathway. The biogenesis of autophagosomes initiates with the phosphorylation and dephosphorylation of the ULK1 complex (ULK1/2, FIP200, Atg13, and Atg101), followed by the activation of the Vps34 lipid kinase complex (VPS34, Atg14L, BECN1, VPS15) which generates PI(3)P. This lipid recruits the proteins ZFYVE and WIPI, locating the Atg5-12-16L complex, and inducing LC3 protein processing by adding a hydrophobic tail that allows it to anchor to the lipidic membranes. The activity of these proteins’ complex generates a deformation in the membranes that ends with the formation of an autophagosome. Finally, the autophagosomes fuse with other endosomes and lysosomes to acquire the degradative characteristics [14]. 

Initially, autophagy was considered a bulk degradation pathway where the entrapped materials were degraded non-specifically. Currently, we know that several recognition molecules or adapters mediate incorporation of material into autophagosomes. Among them, NCK, p62/sequestosome1 (SQSTM1), or NDP52 recognize molecules such as ubiquitin bound to proteins and direct its degradation (reviewed in Ref. [15]). Aged or damaged organelles are also specifically degraded by autophagy. In the context of aging, it is particularly important to mention the participation of autophagy in the maintenance of mitochondrial homeostasis. In the cell, there exist several pathways for targeting mitochondria to autophagic degradation, a process called mitophagy. In the normal metabolism of mitochondria, low levels of ROS are produced. These low levels have a physiological role, whereas high levels of ROS can oxidize nucleic acids, lipids, and proteins leading to cellular dysfunction and programmed cell death [16]. Thus, it is important to clear damaged mitochondria because they can trigger programmed cell death [17], inflammation, and aging [18], and may also participate in many pathophysiological processes [19]. Currently, evidence indicates that Pink1 and Parkin [20,21] or FKBP38, in a Parkin-independent fashion [22], could recognize dysfunctional mitochondria, thus promoting their degradation in a process called mitophagy. In the regulation of mitophagy, the most studied signal is the system Pink1/Parkin. This mechanism involves the ubiquitination of specific mitochondrial domains by Parkin as a consequence of Pink1 activation when mitochondria is depolarized. After ubiquitination, the receptors p62/SQSTM1, NBR1, and optineurin recruit LC3 via their LC3-interacting region (LIR) [23,24,25,26]. In the case of the mitophagy that depends on FKBP38, the recruitment of LC3 occurs by direct binding to a LIR domain present in the FKBP38 protein, [22]. Similar to the FKBP38-dependent mechanism, a group of transmembrane receptors anchored to the mitochondrial membrane could recruit LC3 using a special LIR domain regulated by phosphorylation. This group includes Bnip3 and Bnip3L/Nix, FUNC1, and Bcl2L13/Bcl-Rambo [27]. In summary, the different forms of autophagy, including very specific forms such as mitophagy, play a key role in controlling cellular damage, thus preventing further deterioration processes.

Besides the homeostatic functions, the autophagic pathway acts as a defence mechanism against intracellular pathogens. Xenophagy, a specialized type of autophagy, is a pathway able to recognize pathogens in the cytoplasm or within damaged phagosomes employing adaptor molecules [28,29,30]. Some pathogens have evolved strategies to evade degradation after phagocytosis. This strategy includes blockage of phagosome maturation, generation of small piercings into phagosomal membranes or breaking down of the phagosome, and escape to the cytosol [31]. The autophagic pathway can counteract some of these pathogen strategies. After their escape to the cytoplasm, pathogens are ubiquitinated and receptors like p62 or NDP52 direct them to the autophagic pathway by using LIR and ubiquitin (UB) binding domains [30,32,33,34]. However, escape to the does not need to be recognized by autophagy, since bacteria inside damaged phagosomes/phagolysosomes can also be ubiquitinated. Additionally, vesicles of different maturation stages in the phagocytic pathway present carbohydrates in their lumen that are not normally exposed to the cytoplasm. When phagosomes/phagolysosomes are damaged by the microorganisms, those carbohydrates are exposed, becoming recognisable to specific molecules which collaborate in the autophagic response. Particularly, galectins are recruited to damaged vesicles [32,35] and it has been reported that galectin 8, through binding with NDP52, collaborates in the recruitment of the autophagic machinery to Salmonella-containing vacuoles [32].

**Figure 1:**
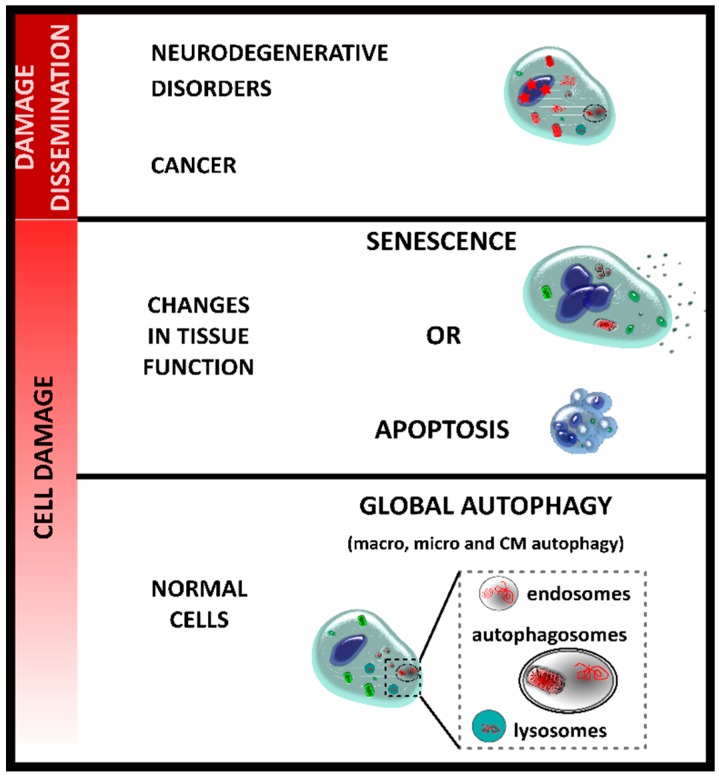
Different stages of cell damage. Autophagy is one of the mechanisms acting as the first barrier against cell damage to avoid the accumulation of non-functional organelles or proteins in the cells. When this mechanism is overburdened, apoptosis or senescence is activated. These mechanisms stop the damaged cells’ dissemination and prevent the apparition of age-related diseases, such as neurodegenerative disorders or cancer. An accumulation of damaged organelles and proteins and changes in the genetic material are observed in these diseases.

Then, global autophagy activation (macro-, micro-, and CM-autophagy, mitophagy, and xenophagy) is the first potent barrier against situations potentially causing harsh cellular damage (Figure 1) [36]. In fact, available data show that a decrease in autophagy activity is associated with the aging process, where accumulation of damaged cells leads to the malfunctioning of tissues in aged organisms [37]. In an interesting work, Garcia-Prat and collaborators showed that basal autophagy is essential to maintain the stem-cell quiescent state in mice. Failure of autophagy in physiologically aged satellite cells, or genetic impairment of autophagy in young cells, causes entry into senescence by loss of proteostasis, increased mitochondrial dysfunction, and oxidative stress, resulting in a decline in the function and number of satellite cells. Re-establishment of autophagy reverses senescence and restores regenerative functions in geriatric satellite cells, indicating that autophagy is necessary to normal regenerative activity of muscle tissue by maintaining satellite stem cell in a quiescent state. 

When the autophagic barrier is overcome, cells have additional mechanisms involved in cellular damage rescue. Proliferative tissues rely on two main strategies to avoid the dissemination of damaged cells, i.e., apoptosis (programmed cell death) or senescence (proliferation arrest) (Figure 1). Apoptosis and senescence appear to be mutually exclusive events and are cell type-dependent. Lymphocytes tend to activate apoptosis, while damaged fibroblasts and epithelial cells mainly undergo senescence [1]. For further reading about apoptosis, we suggest reviews [38,39,40]. This review is mainly focussed in the link between autophagy and senescence in the context of infections.

The term “senescence” was first used by Hayflick and Moorhead in order to name the process where cells stop dividing after a number of culture passages [41]. Current knowledge indicates that this phenomenon is a consequence of telomere shortening and is called replicative senescence. Senescence, however, is a more complex process activated by a multiplicity of stress stimulus connected to cellular damage such as DNA damage, hypoxia, and accumulation of reactive oxygen species (ROS) [42]. All these stimuli seem to converge onto two major signalling pathways, involving two tumour suppressor proteins, the retinoblastoma protein, pRb, and the p53 pathways [43].

Once activated, senescent cells undergo morphological and functional modifications, which include arrest of proliferation, resistance to apoptotic signals, and alteration in gene expression [43]. Phenotypically, senescent cells present flat morphology and increased cell size in vitro. Changes in the chromatin and nuclear morphology have been also observed [44,45]. A key feature is an accumulation of senescence-associated (SA) β-galactosidase, a lysosomal enzyme that has been broadly used as a senescence marker since it becomes easily detected by a conventional staining technique [46,47]. In addition, modifications in gene expression related to down-regulation of genes that promote cell cycle and up-regulation of oncogenes like p16INK4a and p21Waf-Cip1 [43,48] have been detected. Likewise, modifications have been described in an extensive list of genes codifying cytokines. Another characteristic of senescent cells is the secretion of growth-regulating, inflammatory, and tissue remodelling factors [49,50], a process referred to as the senescence-associated secretory phenotype (SASP) [49,51,52]. SASP components actively participate in the senescence process and are used as markers of senescence [53]. For example, interleukin-6 (IL-6) and IL-8 act in an autocrine feedback loop to reinforce the senescence growth arrest [51,53]. In addition, secreted factors from senescent cells may act in a paracrine manner to trigger senescence [54,55]. IL-6, IL-8, and other secreted factors attract immune cells that, together with the increased expression of immune cell-interacting molecules on senescent cells, can lead to immune surveillance and subsequent elimination of senescent cells. In the kidney, some of these factors promote tissue repair by recruiting immune cells that remove damaged components, increase SASP and maintain homeostasis [56,57]. 

Senescence avoids cellular damage spreading by arresting the duplication of cells bearing changes in their genetic information, thus limiting short-term tissue damage [58,59,60,61,62], and preventing tumorigenesis [58,63,64,65]. Furthermore, it has been demonstrated that senescent cells are easily eliminated by the immune system [66]. Dendritic cells, neutrophils, and macrophages were found in the micro-environment of senescent cells [61,67,68]. In a model of mouse hepatic fibrosis, hepatic stellated cells (HCS) undergo senescence and are specifically recognized and eliminated by NK cells [61]. In a similar model, macrophages were able to eliminate HCSs expressing p53 [67].

## 2. Autophagy and Senescence

Based on the consideration that autophagy acts as the first barrier against situations of cellular stress, the traditional point of view states that autophagy prevents senescence (Figure 1) [69,70]. However, when the damage is too extensive to be solved by autophagy, senescence is activated. García-Prat and collaborators showed in 2016 that basal autophagy prevents senescence in muscle stem cells, maintaining the “stemness” and their muscle regenerative nature [37]. In this work, the authors showed that loss of autophagy capacity increases the dysfunction of mitochondria and, as a consequence of an increase in oxidative stress, the senescence program becomes activated. The regenerative capacity of muscle stem cells is restored when autophagy is re-established. Moreover, it has been observed that the high enough accumulation of ROS leads to autophagy inhibition and senescence activation [71,72]. This situation could be reversed by either mTOR inhibition or AMPK activation (two key autophagy regulators), restoring the autophagic activity and, as a consequence, preventing senescence [71,72,73]. Those works are mainly focused on macroautophagy and its role in senescence activation, but CMA has also been related to this process. Senescent fibroblasts and tissues from old organisms show a decrease in transcriptional up-regulation of Hsp70 in response to different stressors [74] and changes have been described in the lipid composition of lysosomal membranes due to aging, as well as accelerated Lamp2A degradation with concomitant protein level diminution [75].

There are some studies at the molecular level about the role of autophagy in senescence regulation. It has been observed that autophagy inhibits senescence by modifying the levels of transcription factor GATA4. This factor initiates the NFKB/NF-kB (nuclear factor kappa-light-chain-enhancer of activated B-cells) transcriptional circuit, involved in SASP generation [76,77]. The autophagic degradation of GATA4 is mediated by the adapter protein p62/SQSTM [77]. After senescence is activated, the interaction between these two proteins decreases and GATA4 accumulates by escaping from degradation.

Nevertheless, a positive role of autophagy in senescence has been reported. In 2009, Young and collaborators showed in a model of oncogene-induced senescence that inhibition of autophagy delayed senescence induced by the oncogene HRASG12V [78]. In the same work, the authors showed that the autophagic pathway is induced in the context of senescence with an up-regulation of the expression levels of several autophagy-related genes such as WIPI-1 and 4, p62, LC3B, Atg7, BNIP3L due to senescence activation. Some years later, Narita and collaborators described the TOR-autophagy spatial coupling compartment (TASCC), a specialized type of autophagy that is able to locally activate mTOR by providing amino acids [79]. This compartment comprises a region of the cell where there is an accumulation of autolysosome-like structures positive for β-galactosidase. Interestingly, mTOR is associated with these structures and such association favours mTOR activation probably due to local amino acids released by the autolysosomes. This activation, in turn, promotes the production of interleukins (IL) 6 and 8, two SASP factors. After this publication, several reports showed that autophagy promotes senescence activation in different models such as fibroblasts in breast cancer by overexpression of CDK (cyclin depending kinases) inhibitors [80] or in radioresistant cancer cell lines by activation of mTOR. Finally, a recent study provided data showing a molecular mechanism of senescence regulation by autophagy. Horikawa and collaborators demonstrated that the p53 inhibitor d133p53 (a p53 isoform) is degraded by autophagy after ubiquitination, which in turn leads to senescence activation [81].

**Figure 2:**
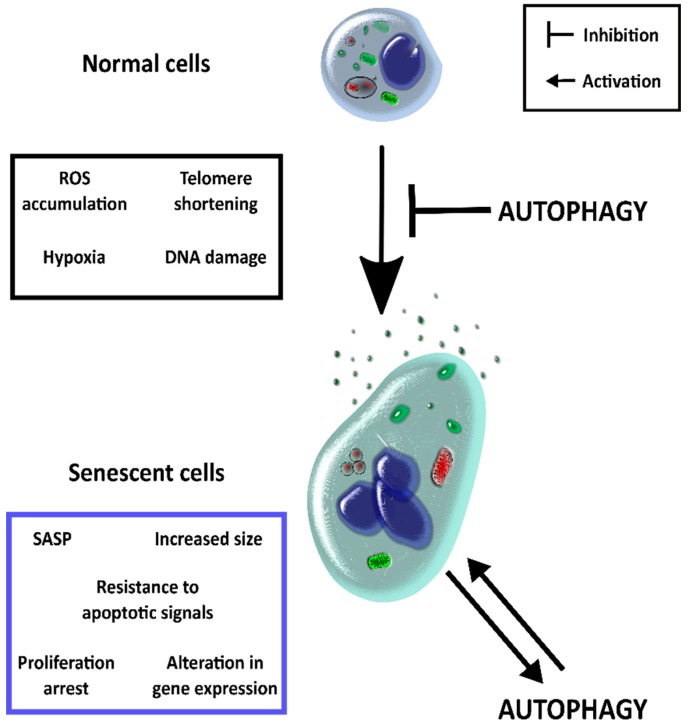
Autophagy has a dual role in senescence activation. There are several factors that stimulate senescence activation. These includes ROS accumulation, hypoxia DNA damage, or telomere shortening. Once activated, senescent cells undergo morphological and functional modifications, which include arrest of proliferation, the resistance to apoptotic signals, size increasing, alteration in gene expression, and activation of SASP. The autophagic pathway could prevent senescence due to its capacity to eliminate potentially dangerous elements generated in aging, such as damaged organelles and proteins; and by collaborating in genome stability. Nevertheless, when senescence is activated, autophagy plays a positive role by autophagic degradation of p53 inhibitors or by increasing the levels of senescent cytokines between others.

Taken together, the existing data show that autophagy could act dually (Figure 2). Firstly, the pathway shows a negative modulation over senescence preventing damage accumulation in the cell. On the other side, when the damage overloads the autophagic capacity, senescence is activated and the autophagic pathway contributes to this activation.

## 3. The Aging of the Immune System

An important consequence of the age-dependent decrease of critical cell functions is the reduced response against pathogen infections. Elderly people display a greater propensity to certain infectious diseases and an increased difficulty to resolve (combat) others, such as seasonal influenza [82] or pneumonia [83]. Related to the immune response, a status of chronic elevated basal inflammation called “inflammaging” characterized by a lower capacity to mount an effective immune response against an infective agent have been described during aging [84]. As part of this process, a gradual deterioration of the immune response, known as immunosenescence, occurs. Moreover, not only T- and B-cells but also neutrophils, macrophages, and dendritic cells are affected (Table 1), leading to the failure of an appropriate innate and adaptive immune response against microorganisms and an attenuated immunological memory to new immunogens [85,86,87,88].

Regarding innate immunity, it has been observed that neutrophils of aged populations show decreased phagocytic activity against opsonized bacteria as well as a marked reduction in superoxide generation [89]. Neutrophils play a key role in the defence against rapidly dividing bacteria, a major cause of infection in the elderly [90,91]. Likewise, macrophages also show diminished phagocytic capability and superoxide production, as well as diminished levels of MHC class II complexes. On the other hand, dendritic cells (DC) display decreased migration capability and alterations in the phagocytic activity of apoptotic cells (reviewed in [89]).

Aging also has an important effect on adaptive immunity. Age-associated immunodeficiency is initially based on the involution of the thymus, the primary organ of T-cell development. Major changes observed in T-cell functions in older adults are associated with the decrease in naïve T-cells and the increase in ineffective memory T-cells [92,93]. Elderly populations possess lower levels of CD4(+) and CD8(+) CD28(+) T-cells often coupled with higher proportions of memory CD8(+) T-cells [94]. Indeed, one of the most important hallmarks of human immune aging is the decrease in the absolute number and percentage of peripheral blood naïve CD8(+) T-cells [94]. A decrease in vaccination efficacy in older adults also correlates with age-associated differences in the responses of CD4(+) and CD8(+) T-cells to the vaccine, associated in turn with variations in DC function [95].

In addition to the phenotype variations of the CD4(+) and CD8(+) T subpopulation, functional changes of T-cells explain most of the age-related pathologies. Lymphocytes of elderly organisms are already in an activated state, unable to be up-regulated by external stimulation [96]. The most affected T-cell functions are clonal expansion and cytokine production, particularly IL-2 [97]. Additionally, a reduced proliferative activity of these cells by senescence activation is observed. T-cells engage the p38MAPK inhibitory signalling pathway in an apparently novel non-canonical mechanism [98]. So, by using small-molecule inhibitors [99] or specific inhibitory shRNA [100], it was observed that p38MAPK inhibition reconstituted proliferation and telomerase activity in T-cells after activation. Senescent T-cells also show high levels of intracellular granules containing the cytotoxic proteins perforin and granzyme B [101], and secrete high levels of inflammatory cytokines such as interferon (IFN)-γ and TNF-α after short-term activation [102]. Besides the age-related thymus regression, the lifetime’s accumulated exposure to infectious agents, autoantigens, and cancer antigens may also induce T-cells replicative senescence and clonal exhaustion, a concept well-established in the case of chronic infections.

An increase in double negative (DN), IgD(−) CD27(−) B cells in elderly populations has been observed that might be an exhausted pool of memory B cells [103]. These cells also have higher basal levels of IL-10 and TNF-α production.

Autophagy dysfunction (macro-, micro-, and CM-autophagy), characteristic of aged organisms [104], contributes to immunosenescence progression. Immune cells with autophagy deficiency also display features of senescence, and in agreement with these data, T-cells and macrophages show decreased autophagy when they are aged.

## 4. Senescence and Chronic Infections

### 4.1. Bacterial Infections and Senescence

*Pseudomonas aeruginosa* is an opportunistic pathogen able to cause infection in cornea and wounds, as well as obstructive respiratory disease and cystic fibrosis [105,106]. Cystic fibrosis is a chronic, asymptomatic disease related to a change in salt concentration due to a failure in the cystic fibrosis transmembrane conductance regulator (CFTR) [107,108]. With the enlargement of the lifetime of patients due to early specific treatment, the chronic infectious disease of the lung has emerged as the main mortality cause in cystic fibrosis patients [109]. The pathogenesis of *P. aeruginosa* is due to a battery of toxins that cause many effects. 

One of the most important toxins is pyocyanin [110], which produces several effects such as apoptosis induction [111], reduction in ciliary movement and sputum velocity in trachea [112,113], change in the production of immune mediators [114,115], and abnormal characteristics and cytotoxicity in skin explants [116] of infected people. Another important effect shown to be caused by pyocyanin is the induction of oxidative stress in epithelial and endothelial cells [117,118]. The induction is moderate but persistent, leading to a senescent phenotype [119]. In this case, the activation of senescence follows the Erk/p38MAPK pathway [108]. Furthermore, pyocyanin is also able to activate the autophagic pathway, which seems not to be related to oxidative stress [120]. Unfortunately, it is not possible to correlate the effect of pyocyanin on autophagy with studies focused on senescence because the experimental conditions are different [108,119,120]. A deeper study is necessary in order to know if there is a relationship between the effect of pyocyanin on autophagy and senescence. Some strategies are to monitoring autophagy and senescence in parallel on pyocyanin-treated cells by prolonged time and use of drugs that modulate autophagy to see the effect of autophagy activation/inhibition on senescence. 

On the other hand, it has been recently observed that epithelial cells of CF patients present an impaired autophagic response with overproduction of ROS and accumulation of aggresomes [121]. Indeed, an interesting study would be to analyse the effect of pyocyanin in normal cells or cells with mutations in the CFTR regarding the senescence phenotype in the absence of an autophagic response. In CF patients, the induction of senescence by *P. aeruginosa* in the airways might be particularly important for chronic infection since senescence activation abrogates the normal desquamation process of airway epithelia, thus allowing bacterial adhesion. 

Indeed, bacteria take advantage in several ways of senescence activation. It has been proposed that reactivation of *Mycobacterium tuberculosis* (Mtb) infection in aged individuals may be, in part, due to senescence or immune exhaustion of T-cells. In aging, T cells expression levels of receptor KLRG1, a receptor that inhibits T-cell function, is increased. Employing a KLRG1-KO mouse model, increased bacterial survival has been demonstrated [122]. Interestingly, the authors proposed that immunosenescence plays a role in the age-associated reactivation of tuberculosis and that KLRG1 is an important participant in the process. Other observations indicate a rapid loss of Mtb-specific CD4+T cells in HIV-infected subjects with active tuberculosis, which may be explained by the particularly high susceptibility of these patients to the HIV-related immune damage and increased mortality [123]. In addition, it has been also shown that co-infection of *Mtb* with HIV contributes to chronic immune activation associated to senescence with functionally altered CD8+ T cells [124,125]. The co-infection process results in an increased HIV viremia with a concomitant decrease in the CD4/CD8 T-cell ratio, leading to suboptimal immune responses. The senescent CD8+ T-cells presented increased levels of CD57 and CD38 with a concomitant decrease of co-stimulatory markers. Indeed, the levels of intracellular IFN-γ, granzyme B, and perforin were diminished in CD8+ T-cells of HIV/ Mtb co-infected patients.

In the case of Mtb infection, it is clear that autophagy has a protective role for the cells against the pathogen, representing an effective antimicrobial response. However, it has also been shown that autophagy may exert inflammation modulation in the host to avoid adverse effects (reviewed by Khan and Jagannath, 2017 [126]). On the other hand, cumulative evidence indicates that several bacterial factors modulate certain components of the autophagic machinery to disrupt the proper functioning of this pathway, but the impact of this disruption on immunosenescence activation has not be addressed to date. One of the most studied *Mycobacterium* factors is the toxin ESAT-6. Several functions have been described for this toxin, but particularly interesting is the inhibition of the maturation of phagosomes/autophagosomes [30,127]. On the other hand, autophagy inducers, such as rapamycin and IFN-γ, revert the inhibition in the maturation produced by *Mycobacterium* [128]. It is possible that blocking of autophagosomal maturation could predispose cells to activation of senescence, and the use of positive modulators prevents the senescence activation. These data could afford the development of new therapeutic strategies against tuberculosis.

Otherwise, a problem to take into account is the chronic and persistent antigenic stimulation, such as bacterial chronic or latent infections that may cause T-cells’ late differentiation or exhaustion. One typical example of this situation may be the chronic infection caused by *Helicobacter pylori*. Curiously, in line with previous examples, *H. pylori* is able to activate senescence and to inhibit the maturation of autophagosomes [129,130,131]. It has been described that CagA toxin activates senescence by up-regulating p21 in an ERK-dependent manner [129]. VacA, another *Helicobacter* toxin, activates autophagy [132] but by inhibition of the maturation of autophagosomes. Interestingly, besides the effect on autophagy, VacA causes mitochondrial depolarization [133] and the disruption of autophagy is accompanied by an elevation of ROS levels [134]. It would be interesting to study whether those effects could collaborate in senescence activation.

Available evidence points to the autophagic pathway as a good candidate to be responsible for senescence activation in chronic infection. This hypothesis is based on the fact that autophagic flux is blocked in the majority of the examples mentioned above, a situation that predisposes infected cells to damage accumulation. Indeed, further studies are necessary to corroborate this hypothesis.

### 4.2. The Chronic Trypanosoma Cruzi Infection

The intracellular protozoan parasite *Trypanosoma cruzi* is the causative agent of Chagas disease, a chronic human infection endemic in Latin-American countries and currently extended to other non-endemic places such as USA, Australia, and Spain. The infection is mainly transmitted by contact with the faeces of an insect vector belonging to the Reduviidae family or by blood transfusion and organs transplants. Parasites invade and replicate in different types of cells and expand the infection by the blood until they reach their specific tissue targets [135]. Earlier in the invasion, *T. cruzi* interacts with endocytic and autophagic pathways and the pre-induction of autophagy benefits the infection by increasing host cell colonization [136]. However, the maturation of autophagosomes was demonstrated to be impaired in infected cells [137]. The chronic disease, present in around 30% of the infected population, is characterized by a persistent inflammatory reaction and destruction of host cells, affecting mainly the peripheral autonomous nervous system of the gastrointestinal tract, the heart muscle, and the cardiac nerves. Clinical symptoms are related to the development of megaesophagus, megacolon, or cardiomegaly associated with progressive and untreatable heart failure [138]. Successful persistence of parasites in muscle cells and neurons is due to the low efficacy of the currently available pharmacological treatments [139] and a dysfunctional immune response by the host. Similar to autoimmune diseases, Chagas disease proceeds with lymphopenia and signs of T-cell senescence. In *T. cruzi*-infected patients, T-cells CD8(+) and CD4(+) present markers of immunosenescence and show an exhausted functional phenotype with diminished production of INF-gamma and IL-2 [140,141,142]. The low frequency of INF-gamma producing CD8(+) T-cells and INF-gamma producing CD4(+) T-cells specific for *T. cruzi* inversely correlates with disease severity [140,141,142]. On the other hand, recent studies have also shown that the thymus is damaged during *T. cruzi* infection and, therefore, lower numbers of conventional thymic T-cell emigrants and Tregs (regulatory T-lymphocytes) reach the peripheral organs. Reduced T-cell renewal along the infection leads to an exacerbated adaptive immune response similar to what has been previously described in other classical autoimmune diseases [143]. Therefore, immunosenescence and autoimmunity are key processes in the Chagas pathology that prevail in about 30% of the infected population, allowing pathogen persistence and the generation of the typical clinical symptoms of the disease.

Together with the evasion of the immune system, *T. cruzi* can also avoid autophagic intracellular degradation through impairment of autophagosome maturation. Blockage of autophagy contributes, like cellular stress protection, to senescence activation. One of the factors leading to parasite persistence is the low efficacy of current treatments [139]. Exploring the roles of autophagy and senescence in Chagas disease could open the door to new therapeutic strategies.

### 4.3. Persistent Human Cytomegalovirus Antigenic Stimulation and Immunosenescense: Possible Role for the Autophagy Pathway

Paradigmatically, persistent viral infections represent the most evident cause of immunosenescence. Contrary to acute infections, persistent infections have a more prolonged duration, mainly because the immune system fails to clear the viral agent which usually resides inside certain cell types (e.g., immune cells, neuronal cells, and epithelial cells). This kind of infection alternates between both productive and silent infection without rapidly killing or even producing excessive damage to the host cells. Varicella-zoster virus, measles virus, HIV-1, HHS-6, HHS-7, HSV-1, HSV-2, and Human Cytomegalovirus (HCMV) are examples of viruses that cause typical persistent infections and whose relation to human T-cell aging has been reviewed in Ref. [144].

Among the above-mentioned viral agents, HCMV merits a more detailed analysis since it causes a unique effect on immunosenescence. HCMV is a viral pathogen belonging to the Herpesviridae family. Clinically, the disease caused by HCMV infections is associated with immunocompromised situations. In new-borns, HCMV infection is associated with deafness, mental retardation, and death, while patients with AIDS often suffer retinitis, pneumonia, or gastrointestinal inflammation by the presence of HCMV. In transplanted patients, the HCMV disease is associated with an increased rate of graft rejection. Certainly, HCMV reactivates intermittently during the host’s lifetime, arguing that sustained antigenic stimulation is the main cause of the virus-enhanced immunosenescence detected in the elderly. However, studies conducted in young children infected with HCMV show similar T-cell imbalance with an increase in memory CD8(+) T-cells and a decrease in the pool of circulating naïve T-cells suggesting a mechanism of immunosenescence independently of the aging process [145,146,147]. Thus, it is conceivable that such imbalance would be deleterious with a decreased overall survival in the elderly associated with the viral infection, a hypothesis that has been confirmed by epidemiological studies [148,149]. Additionally, anti-HCMV IgG titres in aged individuals have been correlated with lower antibody responses to influenza virus [150,151,152]. However, other studies observed the contrary [153,154], so, to date, the relationship between HCMV infections and accelerated immunosenescence remains controversial. Recently, Redeker et al. hypothesized that the observed discrepancies might be related to the variability in the infectious dose of HCMV occurring in real life and they conducted a study to specifically address the role of the infectious dose on the contribution of HCMV to accelerated immune senescence using a mouse model. They showed that the viral inoculum size determines the degree of HCMV-induced immune alterations in lifelong infection. Furthermore, they demonstrated that infection alone caused by a high viral dose reduces newly generated CD8(+) T-cell responses to heterologous super-infection [155].

Given that autophagy is involved in innate and adaptive immunity and it has been described as an antiviral mechanism, it is not surprising that viruses encode proteins to counteract this process, especially in herpesviruses, which are highly adapted to their hosts. HCMV belongs to the group of herpesviruses which subverts autophagy for its own benefit. In this field, Dr. Esclatine´s group and others have reported that HCMV stimulates autophagy early after fibroblasts infection and that components of the viral particles, such as viral DNA, are sufficient to trigger this mechanism [156,157]. However, later on, the autophagic flux is blocked by the action of the viral proteins TRS1 and IRS1 [158]. Several viral proteins able to modulate autophagy act by interacting with BECN1, thus disrupting autophagy, either at the autophagosome formation or at the maturation step [159]. The Nef protein of HIV-1, [160], the M2 protein of influenza virus [161] and ICP34.5 of HSV-1 [162] suppress autophagosome maturation into autolysosomes, likewise via their interaction with BECN1. Mouna and collaborators [158] found that the coiled-coil domain (CCD) in BECN1, a universal oligomerization domain, is the binding domain of IRS1. Notably, this CCD domain allows ATG14 and UVRAG (UV radiation resistance associated) to interact with BECN1 in two distinct PtdIns3K-containing complexes that function differentially in autophagosome formation and in the maturation of the endosome and the autophagosome [163]. Their results suggest that individually, IRS1 and TRS1 may block autophagosome biogenesis through the PtdIns3K-BECN1-ATG14 complex, and co-expression of these two proteins may block the autophagy maturation process through the PtdIns3K BECN1-UVRAG complex. Interestingly, the authors have shown that chemical induction of autophagy enhanced HCMV production, while treatment with SPAUTIN (Specific and Potent Autophagy Inhibitor) produced a decrease in viral titres. Therefore, it is likely that HCMV uses autophagic proteins or membranes for its propagation. Indeed, understanding which steps of the herpes virus cycle are facilitated by autophagy should be a major research focus for Herpes virologists.

To date, based on reported studies, HCMV-associated immunosenescence and HCMV–autophagy interaction appear to be independent, but future prospective longitudinal studies will be required to further delineate the contribution of HCMV-induced autophagy inhibition to immunosenescence and to determine their impact in the process of aging. However, taken together, we hypothesize that the autophagy-inhibited status of HCMV-infected cells might contribute to senescence of these cells in addition to the immunosenescence induced by the persistence of circulating viral antigens.

## 5. Concluding Remarks

Senescence is induced as a consequence of cellular damage accumulation, with the extent of activation directly depending on a fine-tuned balance between cellular conditions generating damage and those involved in counteracting them. The autophagic pathway plays a key role in preventing cell damage accumulation, however, the aging process leads to a decrease in autophagy capacity, and therefore also its effectiveness. In this context, senescence activation shows a more preponderant protective role. 

The immune system does not escape from aging effects and displays senescence characteristics in aged individuals. Immunosenescence refers to the state of dysregulated immune function that contributes to the increased susceptibility to infections, autoimmune diseases, or cancer. Aged individuals are predisposed to more severe symptoms from certain infections and they do not mount an effective immune response upon vaccination. In general, aged populations fail to generate an appropriate innate and adaptive immune response against microorganisms, thus it becomes clear that senescence is involved in this failure. 

**Figure 3:**
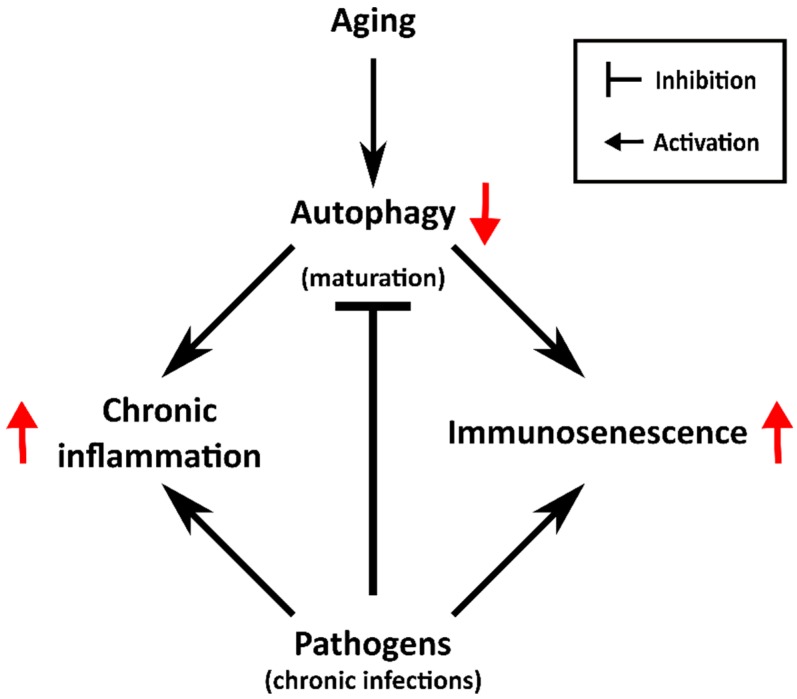
One of the consequences of the aging process is the diminution of autophagic capacity. Consequently, this diminution predisposes the individual to accumulation of cellular damage and senescence activation. In the immune system, this process is called immunosenescence and is accompanied by chronic inflammation. Several pathogens that induce chronic infections are able to activate or accelerate the immunosenescence process and in this way, their elimination by the host tends to be less effective.

Besides the normal occurrence of immunosenescence, several pathogen microorganisms accelerate the activation of senescence and predisposal to premature immunosenescence (Figure 3). For instance, hosts infected with bacteria such as *P. aeuruginosa*, *M. tuberculosis*, or *H. pylori,* some viruses, including HCMV, or the parasite *T. cruzi*, show characteristics of immunosenescence (Table 2).

A common issue of all of these pathogens is that they are able to generate chronic infections. In each of these, regardless of the fact that the host is faced with the same antigen several times during its lifetime, the immune response is inefficient. Furthermore, data shows that this condition generates an immune exhaustion and immunosenescence seems to be the major causative factor offering the pathogens an extra advantage since their elimination by the host tends to be even less effective.

Nevertheless, there is also strong evidence that senescence activation does not necessarily depend on a prolonged time of exposure of antigens to the immune system. HCMV is able to activate senescence in children regardless of infection duration. Besides, there are some factors (toxins) that can directly activate senescence, such as pyocyanin from *P. aureginosa* or CagA from *H. pylori*.

Interestingly, a common characteristic of chronic infections is the autophagy blockage that usually occurs during autophagosome maturation, representing a factor that could contribute to or accelerate immunosenescence activation since it predisposes cells to damage accumulation.

The majority of the examples here focus on the macroautophagy, which may be due to the relationship between xenophagy and pathogens. Nevertheless, there are examples of CMA–pathogen interactions. *Salmonella enterica* recruits Lamp2A and Hsp70 to its vacuole, favouring nutrient acquisition by the bacteria [164], but the effect of this sequestration over cell homeostasis has not been studied yet. Unfortunately, we currently lack works focusing on the role of the autophagic pathway in senescence activation when cells are infected with pathogens, or if the direct activation of senescence by pathogens affects autophagy. Appropriate experiments must be conducted to specifically delineate the role of autophagy (use of modulation drugs, knock out cells for Atg proteins, etc.) in senescence regulation, and/or the opposite direction in infected models. One interesting question to solve is if there is greater damage accumulation in cells when autophagy is blocked by pathogens, and if this effect could produce or collaborate with other factors in the senescence response.

Then, deeper exploration to elucidate whether the activation of senescence in chronic infection is a consequence of autophagy impairment produced by pathogens to avoid degradation or, alternatively, whether it is a mechanism employed by the host to diminish infection spreading when the degradation of the pathogens has been halted. This exploration is needed to further understand the infection–autophagy–senescence relationship. With the available data, we hypothesize that chronic infections induce senescence with similar characteristics of aging, i.e., increase of inflammatory state and autophagy inhibition (Figure 3).

In some cases, pathogens have specific tools such as toxins that reinforce the effect of senescence activation. This evidence denotes an important role of senescence in the survival of the pathogen. The understanding of immune cells’ modifications in normal situations or in the context of chronic infections should contribute to the design and development of novel therapeutic strategies to prevent and treat such alterations.

## Figures and Tables

**Table 1:** Main effect of senescence over immune system.

Type of Immunity	Cell Type	Senescent Phenotype	
Innate immunity	Neutrophils	Phagocytic activity Superoxide generation	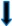
	Macrophages	Phagocytic capabilities Superoxide production MHC Levels	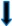
	Dendritic cells	Migration capabilities	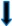
		Alterations in phagocytic activity of apoptotic cells	
Adaptive immunity	T-cells	CD4(+) and CD8(+) CD28(+)	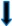
		Proportion CD8(+) CD28(+)	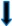
		Blood naïve CD8(+)	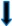
		Proliferative activity	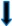
	B-cells	IgD(−) CD27(−)	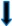
		Basal levels of IL-10 and TNF-α	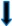

**Table 2:** Effect of pathogens on autophagy and senescence.

Pathogen	Autophagy	Senescence
*Pseudomonas aeruginosa*	Activated (pyocyanin)	Activated (pyocyanin)
*Mycobacterium tuberculosis*	Blocked autophagic flux (ESAT-6)	Activated (immunosenescence)
*Helicobacter pylori*	Blocked autophagic flux (VacA)	Activated (CagA)
*Trypanosoma cruzi*	Activated Blocked autophagic flux	Activated
HCMV	Activated Blocked autophagic flux (TRS1 and IRS1)	Activated (immunosenescence)

## References

[1] Vicencio J.M., Galluzzi L., Tajeddine N., Ortiz C., Criollo A., Tasdemir E., Morselli E., Ben Younes A., Maiuri M.C., Lavandero S. (2008). Senescence, apoptosis or autophagy?. Gerontology.

[2] Dikic I. (2017). Proteasomal and Autophagic Degradation Systems. Annu. Rev. Biochem..

[3] Kaushik S., Cuervo A. (2013). Selective autophagy in cellular quality control. Protein Quality Control in Neurodegenerative Diseases.

[4] Matsui A., Kamada Y., Matsuura A. (2013). The Role of Autophagy in Genome Stability through Suppression of Abnormal Mitosis under Starvation. PLoS Genet..

[5] Zhao Z., Oh S., Li D., Ni D., Pirooz S.D., Lee J.H., Yang S., Lee J.Y., Ghozalli I., Costanzo V. (2012). A Dual Role for UVRAG in Maintaining Chromosomal Stability Independent of Autophagy. Dev. Cell.

[6] Cuervo A.M., Wong E. (2014). Chaperone-mediated autophagy: Roles in disease and aging. Cell Res..

[7] Li W., Nie T., Xu H., Yang J., Yang Q., Mao Z. (2018). Chaperone-mediated autophagy: Advances from bench to bedside. Neurobiol. Dis..

[8] Salvador N., Aguado C., Horst M., Knecht E. (2000). Import of a cytosolic protein into lysosomes by chaperone-mediated autophagy depends on its folding state. J. Biol. Chem..

[9] Galluzzi L., Baehrecke E.H., Ballabio A., Boya P., Bravo-San Pedro J.M., Cecconi F., Choi A.M., Chu C.T., Codogno P., Colombo M.I. (2017). Molecular definitions of autophagy and related processes. EMBO J..

[10] Tekirdag K., Cuervo A.M. (2018). Chaperone-mediated autophagy and endosomal microautophagy: Joint by a chaperone. J. Biol. Chem..

[11] Sahu R., Kaushik S., Clement C.C., Cannizzo E.S., Scharf B., Follenzi A., Potolicchio I., Nieves E., Cuervo A.M., Santambrogio L. (2011). Microautophagy of Cytosolic Proteins by Late Endosomes. Dev. Cell.

[12] Koga H., Martinez-Vicente M., MacIan F., Verkhusha V.V., Cuervo A.M. (2011). A photoconvertible fluorescent reporter to track chaperone-mediated autophagy. Nat. Commun..

[13] Reggiori F., Ungermann C. (2017). Autophagosome Maturation and Fusion. J. Mol. Biol..

[14] Fader C.M., Aguilera M.O., Colombo M.I. (2015). Autophagy response: Manipulating the mTOR-controlled machinery by amino acids and pathogens. Amino Acids.

[15] Johansen T., Lamark T. (2011). Selective autophagy mediated by autophagic adapter proteins. Autophagy.

[16] Hamanaka R.B., Chandel N.S. (2010). Mitochondrial reactive oxygen species regulate cellular signaling and dictate biological outcomes. Trends Biochem. Sci..

[17] Green D.R., Kroemer G. (2004). The Pathophysiology of Mitochondrial Cell Death. Science.

[18] Green D.R., Galluzzi L., Kroemer G. (2011). Mitochondria and the Autophagy-Inflammation-Cell Death Axis in Organismal Aging. Science.

[19] Chan D.C. (2006). Mitochondria: Dynamic Organelles in Disease, Aging, and Development. Cell.

[20] Yoo S.-M., Jung Y.-K. (2018). A Molecular Approach to Mitophagy and Mitochondrial Dynamics. Mol. Cells.

[21] Jin S.M., Youle R.J. (2012). PINK1- and Parkin-mediated mitophagy at a glance. J. Cell Sci..

[22] Bhujabal Z., Birgisdottir Å.B., Sjøttem E., Brenne H.B., Øvervatn A., Habisov S., Kirkin V., Lamark T., Johansen T. (2017). FKBP8 recruits LC3A to mediate Parkin-independent mitophagy. EMBO Rep..

[23] Sarraf S.A., Raman M., Guarani-Pereira V., Sowa M.E., Huttlin E.L., Gygi S.P., Harper J.W. (2013). Landscape of the PARKIN-dependent ubiquitylome in response to mitochondrial depolarization. Nature.

[24] Chan N.C., Salazar A.M., Pham A.H., Sweredoski M.J., Kolawa N.J., Graham R.L.J., Hess S., Chan D.C. (2011). Broad activation of the ubiquitin-proteasome system by Parkin is critical for mitophagy. Hum. Mol. Genet..

[25] Wong Y.C., Holzbaur E.L.F. (2014). Optineurin is an autophagy receptor for damaged mitochondria in parkin-mediated mitophagy that is disrupted by an ALS-linked mutation. Proc. Natl. Acad. Sci. USA.

[26] Narendra D., Tanaka A., Suen D.F., Youle R.J. (2008). Parkin is recruited selectively to impaired mitochondria and promotes their autophagy. J. Cell Biol..

[27] Hamacher-Brady A., Brady N.R. (2016). Mitophagy programs: Mechanisms and physiological implications of mitochondrial targeting by autophagy. Cell. Mol. Life Sci..

[28] Kim B.W., Kwon D.H., Song H.K. (2016). Structure biology of selective autophagy receptors. BMB Rep..

[29] Svenning S., Johansen T. (2013). Selective autophagy. Essays Biochem..

[30] Kwon D.H., Song H.K. (2018). A Structural View of Xenophagy, a Battle between Host and Microbes. Mol. Cells.

[31] Flannagan R.S., Cosío G., Grinstein S. (2009). Antimicrobial mechanisms of phagocytes and bacterial evasion strategies. Nat. Rev. Microbiol..

[32] Thurston T.L.M., Ryzhakov G., Bloor S., von Muhlinen N., Randow F. (2009). The TBK1 adaptor and autophagy receptor NDP52 restricts the proliferation of ubiquitin-coated bacteria. Nat. Immunol..

[33] Levine B., Mizushima N., Virgin H.W. (2011). Autophagy in immunity and inflammation. Nature.

[34] Perrin A.J., Jiang X., Birmingham C.L., So N.S., Brumell J.H. (2004). Recognition of bacteria in the cytosol of mammalian cells by the ubiquitin system. Curr. Biol..

[35] Mansilla Pareja M.E., Colombo M.I. (2013). Autophagic clearance of bacterial pathogens: Molecular recognition of intracellular microorganisms. Front. Cell. Infect. Microbiol..

[36] Kroemer G., Mariño G., Levine B. (2010). Autophagy and the Integrated Stress Response. Mol. Cell.

[37] García-Prat L., Martínez-Vicente M., Perdiguero E., Ortet L., Rodríguez-Ubreva J., Rebollo E., Ruiz-Bonilla V., Gutarra S., Ballestar E., Serrano A.L. (2016). Autophagy maintains stemness by preventing senescence. Nature.

[38] O’Brien M.A., Kirby R. (2008). Apoptosis: A review of pro-apoptotic and anti-apoptotic pathways and dysregulation in disease. J. Vet. Emerg. Crit. Care.

[39] Elmore S. (2007). Apoptosis: A Review of Programmed Cell Death. Toxicol. Pathol..

[40] Hongmei Z. (2012). Extrinsic and Intrinsic Apoptosis Signal Pathway Review. Apoptosis and Medicine.

[41] Hayflick L., Moorhead P.S. (1961). The serial cultivation of human diploid strains. Exp. Cell Res..

[42] Chen Q., Fischer A., Reagan J.D., Yan L.J., Ames B.N. (1995). Oxidative DNA damage and senescence of human diploid fibroblast cells. Proc. Natl. Acad. Sci. USA.

[43] Campisi J., D’Adda Di Fagagna F. (2007). Cellular senescence: When bad things happen to good cells. Nat. Rev. Mol. Cell Biol..

[44] Narita M., Nũnez S., Heard E., Narita M., Lin A.W., Hearn S.A., Spector D.L., Hannon G.J., Lowe S.W. (2003). Rb-mediated heterochromatin formation and silencing of E2F target genes during cellular senescence. Cell.

[45] Rai T.S., Adams P.D. (2012). Lessons from senescence: Chromatin maintenance in non-proliferating cells. BBA-Gene Regul. Mech..

[46] Debacq-Chainiaux F., Erusalimsky J.D., Campisi J., Toussaint O. (2009). Protocols to detect senescence-associated beta-galactosidase (SA-βgal) activity, a biomarker of senescent cells in culture and in vivo. Nat. Protoc..

[47] Dimri G.P., Lee X., Basile G., Acosta M., Scott G., Roskelley C., Medrano E.E., Linskens M., Rubelj I., Pereira-Smith O. (1995). A biomarker that identifies senescent human cells in culture and in aging skin in vivo. Proc. Natl. Acad. Sci. USA.

[48] Collado M., Blasco M.A., Serrano M. (2007). Cellular Senescence in Cancer and Aging. Cell.

[49] Coppé J.-P., Patil C.K., Rodier F., Sun Y., Muñoz D.P., Goldstein J., Nelson P.S., Desprez P.-Y., Campisi J. (2008). Senescence-Associated Secretory Phenotypes Reveal Cell-Nonautonomous Functions of Oncogenic RAS and the p53 Tumor Suppressor. PLoS Biol..

[50] Freund A., Orjalo A.V., Desprez P.Y., Campisi J. (2010). Inflammatory Networks during Cellular Senescence: Causes and Consequences. Trends Mol. Med..

[51] Kuilman T., Peeper D.S. (2009). Senescence-messaging secretome: SMS-ing cellular stress. Nat. Rev. Cancer.

[52] Byun H.O., Lee Y.K., Kim J.M., Yoon G. (2015). From cell senescence to age-related diseases: Differential mechanisms of action of senescence-associated secretory phenotypes. BMB Rep..

[53] Kortlever R.M., Higgins P.J., Bernards R. (2006). Plasminogen activator inhibitor-1 is a critical downstream target of p53 in the induction of replicative senescence. Nat. Cell Biol..

[54] Krtolica A., Parrinello S., Lockett S., Desprez P.-Y., Campisi J. (2001). Senescent fibroblasts promote epithelial cell growth and tumorigenesis: A link between cancer and aging. Proc. Natl. Acad. Sci. USA.

[55] Wajapeyee N., Serra R.W., Zhu X., Mahalingam M., Green M.R. (2008). Oncogenic BRAF Induces Senescence and Apoptosis through Pathways Mediated by the Secreted Protein IGFBP7. Cell.

[56] Casiraghi F., Perico N., Cortinovis M., Remuzzi G. (2016). Mesenchymal stromal cells in renal transplantation: Opportunities and challenges. Nat. Rev. Nephrol..

[57] Weisheit C.K., Engel D.R., Kurts C. (2015). Dendritic cells and macrophages: Sentinels in the kidney. Clin. J. Am. Soc. Nephrol..

[58] Chen Z., Trotman L.C., Shaffer D., Lin H.K., Dotan Z.A., Niki M., Koutcher J.A., Scher H.I., Ludwig T., Gerald W. (2005). Crucial role of p53-dependent cellular senescence in suppression of Pten-deficient tumorigenesis. Nature.

[59] Fitzner B., Müller S., Walther M., Fischer M., Engelmann R., Müller-Hilke B., Pützer B.M., Kreutzer M., Nizze H., Jaster R. (2012). Senescence determines the fate of activated rat pancreatic stellate cells. J. Cell. Mol. Med..

[60] Jun J.I., Lau L.F. (2010). The matricellular protein CCN1 induces fibroblast senescence and restricts fibrosis in cutaneous wound healing. Nat. Cell Biol..

[61] Krizhanovsky V., Yon M., Dickins R.A., Hearn S., Simon J., Miething C., Yee H., Zender L., Lowe S.W. (2008). Senescence of Activated Stellate Cells Limits Liver Fibrosis. Cell.

[62] Pitiyage G.N., Slijepcevic P., Gabrani A., Chianea Y.G., Lim K.P., Prime S.S., Tilakaratne W.M., Fortune F., Parkinson E.K. (2011). Senescent mesenchymal cells accumulate in human fibrosis by a telomere-independent mechanism and ameliorate fibrosis through matrix metalloproteinases. J. Pathol..

[63] Braig M., Lee S., Loddenkemper C., Rudolph C., Peters A.H.F.M., Schlegelberger B., Stein H., Dörken B., Jenuwein T., Schmitt C.A. (2005). Oncogene-induced senescence as an initial barrier in lymphoma development. Nature.

[64] Collado M., Gil J., Efeyan A., Guerra C., Schuhmacher A.J., Barradas M., Benguría A., Zaballos A., Flores J.M., Barbacid M. (2005). Tumour biology: Senescence in premalignant tumours. Nature.

[65] Michaloglou C., Vredeveld L.C.W., Soengas M.S., Denoyelle C., Kuilman T., Van Der Horst C.M.A.M., Majoor D.M., Shay J.W., Mooi W.J., Peeper D.S. (2005). BRAFE600-associated senescence-like cell cycle arrest of human naevi. Nature.

[66] Sagiv A., Krizhanovsky V. (2013). Immunosurveillance of senescent cells: The bright side of the senescence program. Biogerontology.

[67] Lujambio A., Akkari L., Simon J., Grace D., Tschaharganeh D.F., Bolden J.E., Zhao Z., Thapar V., Joyce J.A., Krizhanovsky V. (2013). Non-cell-autonomous tumor suppression by p53. Cell.

[68] Xue W., Zender L., Miething C., Dickins R.A., Hernando E., Krizhanovsky V., Cordon-Cardo C., Lowe S.W. (2007). Senescence and tumour clearance is triggered by p53 restoration in murine liver carcinomas. Nature.

[69] Levine B., Kroemer G. (2008). Autophagy in the Pathogenesis of Disease. Cell.

[70] Rubinsztein D.C., Mariño G., Kroemer G. (2011). Autophagy and aging. Cell.

[71] Han X., Tai H., Wang X., Wang Z., Zhou J., Wei X., Ding Y., Gong H., Mo C., Zhang J. (2016). AMPK activation protects cells from oxidative stress-induced senescence via autophagic flux restoration and intracellular NAD + elevation. Aging Cell.

[72] Tai H., Wang Z., Gong H., Han X., Zhou J., Wang X., Wei X., Ding Y., Huang N., Qin J. (2017). Autophagy impairment with lysosomal and mitochondrial dysfunction is an important characteristic of oxidative stress-induced senescence. Autophagy.

[73] Nopparat C., Sinjanakhom P., Govitrapong P. (2017). Melatonin reverses H_2_O_2_-induced senescence in SH-SY5Y cells by enhancing autophagy via sirtuin 1 deacetylation of the RelA/p65 subunit of NF-κB. J. Pineal Res..

[74] Koga H., Kaushik S., Cuervo A.M. (2011). Protein homeostasis and aging: The importance of exquisite quality control. Ageing Res. Rev..

[75] Kiffin R., Kaushik S., Zeng M., Bandyopadhyay U., Zhang C., Massey A.C., Martinez-Vicente M., Cuervo A.M. (2007). Altered dynamics of the lysosomal receptor for chaperone-mediated autophagy with age. J. Cell Sci..

[76] Kang C., Xu Q., Martin T.D., Li M.Z., Demaria M., Aron L., Lu T., Yankner B.A., Campisi J., Elledge S.J. (2015). The DNA damage response induces inflammation and senescence by inhibiting autophagy of GATA4. Science.

[77] Kang C., Elledge S.J. (2016). How autophagy both activates and inhibits cellular senescence. Autophagy.

[78] Young A.R.J., Narita M., Ferreira M., Kirschner K., Sadaie M., Darot J.F.J., Tavaré S., Arakawa S., Shimizu S., Watt F.M. (2009). Autophagy mediates the mitotic senescence transition. Genes Dev..

[79] Narita M., Young A.R.J., Arakawa S., Samarajiwa S.A., Nakashima T., Yoshida S., Hong S., Berry L.S., Reichelt S., Ferreira M. (2011). Spatial coupling of mTOR and autophagy augments secretory phenotypes. Science.

[80] Capparelli C., Chiavarina B., Whitaker-Menezes D., Pestell T.G., Pestell R.G., Hulit J., Andò S., Howell A., Martinez-Outschoorn U.E., Sotgia F. (2012). CDK inhibitors (p16/p19/p21) induce senescence and autophagy in cancer-associated fibroblasts, tumor growth via paracrine interactions, without an increase in neo-angiogenesis. Cell Cycle.

[81] Horikawa I., Fujita K., Jenkins L.M.M., Hiyoshi Y., Mondal A.M., Vojtesek B., Lane D.P., Appella E., Harris C.C. (2014). Autophagic degradation of the inhibitory p53 isoform δ 133p53α as a regulatory mechanism for p53-mediated senescence. Nat. Commun..

[82] Gross P.A. (1991). Current recommendations for the prevention and treatment of influenza in the older population. Drugs Aging.

[83] Janssens J.-P., Krause K.-H. (2004). Pneumonia in the very old. Lancet Infect. Dis..

[84] Franceschi C., Bonafe M., Valensin S., Olivieri F., De Luca M., Ottviani E., De Benedictis G. (2006). Inflamm-aging: An Evolutionary Perspective on Immunosenescence. Ann. N. Y. Acad. Sci..

[85] Aspinall R., Del Giudice G., Effros R.B., Grubeck-Loebenstein B., Sambhara S. (2007). Challenges for vaccination in the elderly. Immun. Ageing.

[86] Fietta A., Merlini C., De Bernardi P.M., Gandola L., Piccioni P.D., Grassi C. (1993). Non specific immunity in aged healthy subjects and in patients with chronic bronchitis. Aging Milano.

[87] Martín S., Pérez A., Aldecoa C. (2017). Sepsis and Immunosenescence in the Elderly Patient: A Review. Front. Med..

[88] Vardi M., Ghanem-Zoubi N.O., Bitterman H., Abo-Helo N., Yurin V., Weber G., Laor A. (2013). Sepsis in nonagenarians admitted to internal medicine departments: A comparative study of outcomes. QJM.

[89] Weiskopf D., Weinberger B., Grubeck-Loebenstein B. (2009). The aging of the immune system. Transpl. Int..

[90] Butcher S., Chahel H., Lord J.M. (2000). Ageing and the neutrophil: No appetite for killing?. Immunology.

[91] Butcher S.K., Chahal H., Nayak L., Sinclair A., Henriquez N.V., Sapey E., O’Mahony D., Lord J.M. (2001). Senescence in innate immune responses: Reduced neutrophil phagocytic capacity and CD16 expression in elderly humans. J. Leukoc. Biol..

[92] Appay V., Sauce D. (2014). Naive T cells: The crux of cellular immune aging?. Exp. Gerontol..

[93] McElhaney J.E., Effros R.B. (2009). Immunosenescence: What does it mean to health outcomes in older adults?. Curr. Opin. Immunol..

[94] Pawelec G. (2018). Age and immunity: What is “immunosenescence”?. Exp. Gerontol..

[95] Stervbo U., Meier S., Mälzer J.N., Baron U., Bozzetti C., Jürchott K., Nienen M., Olek S., Rachwalik D., Schulz A.R. (2015). Effects of aging on human leukocytes (part I): Immunophenotyping of innate immune cells. Age.

[96] Le Page A., Dupuis G., Larbi A., Witkowski J.M., Fülöp T. (2018). Signal transduction changes in CD4+ and CD8+ T cell subpopulations with aging. Exp. Gerontol..

[97] Larbi A., Dupuis G., Khalil A., Douziech N., Fortin C., Fülöp T. (2006). Differential role of lipid rafts in the functions of CD4+ and CD8+ human T lymphocytes with aging. Cell. Signal..

[98] Akbar A.N., Henson S.M., Lanna A. (2016). Senescence of T Lymphocytes: Implications for Enhancing Human Immunity. Trends Immunol..

[99] Di Mitri D., Azevedo R.I., Henson S.M., Libri V., Riddell N.E., Macaulay R., Kipling D., Soares M.V.D., Battistini L., Akbar A.N. (2011). Reversible Senescence in Human CD4+CD45RA+CD27- Memory T Cells. J. Immunol..

[100] Lanna A., Henson S.M., Escors D., Akbar A.N. (2014). The kinase p38 activated by the metabolic regulator AMPK and scaffold TAB1 drives the senescence of human T cells. Nat. Immunol..

[101] Long H.M., Leese A.M., Chagoury O.L., Connerty S.R., Quarcoopome J., Quinn L.L., Shannon-Lowe C., Rickinson A.B. (2011). Cytotoxic CD4+ T Cell Responses to EBV Contrast with CD8 Responses in Breadth of Lytic Cycle Antigen Choice and in Lytic Cycle Recognition. J. Immunol..

[102] Libri V., Azevedo R.I., Jackson S.E., Di Mitri D., Lachmann R., Fuhrmann S., Vukmanovic-Stejic M., Yong K., Battistini L., Kern F. (2011). Cytomegalovirus infection induces the accumulation of short-lived, multifunctional CD4+ CD45RA+ CD27− T cells: The potential involvement of interleukin-7 in this process. Immunology.

[103] Colonna-Romano G., Bulati M., Aquino A., Pellicanò M., Vitello S., Lio D., Candore G., Caruso C. (2009). A double-negative (IgD-CD27-) B cell population is increased in the peripheral blood of elderly people. Mech. Ageing Dev..

[104] Cuervo A.M. (2008). Autophagy and aging: Keeping that old broom working. Trends Genet..

[105] Bhagirath A.Y., Li Y., Somayajula D., Dadashi M., Badr S., Duan K. (2016). Cystic fibrosis lung environment and *Pseudomonas aeruginosa* infection. BMC Pulm. Med..

[106] Lund-Palau H., Turnbull A.R., Bush A., Bardin E., Cameron L., Soren O., Wierre-Gore N., Alton E.W.F.W., Bundy J.G., Connett G. (2016). *Pseudomonas aeruginosa* infection in cystic fibrosis: Pathophysiological mechanisms and therapeutic approaches. Expert Rev. Respir. Med..

[107] Castellani C., Cuppens H., Macek M., Cassiman J.J., Kerem E., Durie P., Tullis E., Assael B.M., Bombieri C., Brown A. (2008). Consensus on the use and interpretation of cystic fibrosis mutation analysis in clinical practice. J. Cyst. Fibros..

[108] Muller M., Li Z., Maitz P.K.M. (2009). *Pseudomonas* pyocyanin inhibits wound repair by inducing premature cellular senescence: Role for p38 mitogen-activated protein kinase. Burns.

[109] Shepherd R., Cooksley W.G.E., Cooke W.D.D. (1980). Improved growth and clinical, nutritional, and respiratory changes in response to nutritional therapy in cystic fibrosis. J. Pediatr..

[110] Sadikot R.T., Blackwell T.S., Christman J.W., Prince A.S. (2005). Pathogen-host interactions in pseudomonas aeruginosa pneumonia. Am. J. Respir. Crit. Care Med..

[111] Allen L., Dockrell D.H., Pattery T., Lee D.G., Cornelis P., Hellewell P.G., Whyte M.K.B. (2005). Pyocyanin Production by Pseudomonas aeruginosa Induces Neutrophil Apoptosis and Impairs Neutrophil-Mediated Host Defenses In Vivo. J. Immunol..

[112] Munro N.C., Barker A., Rutman A., Taylor G., Watson D., McDonald-Gibson W.J., Towart R., Taylor W.A., Wilson R., Cole P.J. (1989). Effect of pyocyanin and 1-hydroxyphenazine on in vivo tracheal mucus velocity. J. Appl. Physiol..

[113] Wilson R., Sykes D.A., Watson D., Rutman A., Taylor G.W., Cole P.J. (1988). Measurement of *Pseudomonas aeruginosa* phenazine pigments in sputum and assessment of their contribution to sputum sol toxicity for respiratory epithelium. Infect. Immun..

[114] Denning G.M., Wollenweber L.A., Railsback M.A., Cox C.D., Stoll L.L., Bntigan B.E., Britigan B.E. (1998). *Pseudomonas* Pyocyanin Increases Interleukin-8 Expression by Human Airway *Pseudomonas* Pyocyanin Increases Interleukin-8 Expression by Human Airway Epithelial Cells. Infect. Immun..

[115] Muller M., Sorrell T.C. (1991). Production of leukotriene B4 and 5-hydroxyeicosatetraenoic acid by human neutrophils is inhibited by *Pseudomonas aeruginosa* phenazine derivatives. Infect. Immun..

[116] Cruickshank C.N., Lowbury E.J. (1953). The effect of pyocyanin on human skin cells and leucocytes. Br. J. Exp. Pathol..

[117] Muller M. (2002). Pyocyanin induces oxidative stress in human endothelial cells and modulates the glutathione redox cycle. Free Radic. Biol. Med..

[118] O’Malley Y.Q., Abdalla M.Y., McCormick M.L., Reszka K.J., Denning G.M., Britigan B.E., O’Malley Y.Q., Abdalla M.Y., McCormick M.L., Reszka K.J. (2003). Subcellular localization of *Pseudomonas* pyocyanin cytotoxicity in human lung epithelial cells. Am. J. Physiol.-Lung Cell. Mol. Physiol..

[119] Muller M. (2006). Premature cellular senescence induced by pyocyanin, a redox-active *Pseudomonas aeruginosa* toxin. Free Radic. Biol. Med..

[120] Yang Z.S., Ma L.Q., Zhu K., Yan J.Y., Bian L., Zhang K.Q., Zou C.G. (2016). *Pseudomonas* toxin pyocyanin triggers autophagy: Implications for pathoadaptive mutations. Autophagy.

[121] Luciani A., Villella V.R., Esposito S., Brunetti-Pierri N., Medina D., Settembre C., Gavina M., Pulze L., Giardino I., Pettoello-Mantovani M. (2010). Defective CFTR induces aggresome formation and lung inflammation in cystic fibrosis through ROS-mediated autophagy inhibition. Nat. Cell Biol..

[122] Cyktor J.C., Carruthers B., Stromberg P., Flaño E., Pircher H., Turnera J. (2013). Killer cell lectin-like receptor G1 deficiency significantly enhances survival after *Mycobacterium tuberculosis* infection. Infect. Immun..

[123] Geldmacher C., Ngwenyama N., Schuetz A., Petrovas C., Reither K., Heeregrave E.J., Casazza J.P., Ambrozak D.R., Louder M., Ampofo W. (2010). Preferential infection and depletion of *Mycobacterium tuberculosis*—Specific CD4 T cells after HIV-1 infection. J. Exp. Med..

[124] Barathan M., Mohamed R., Vadivelu J., Chang L.Y., Vignesh R., Krishnan J., Sigamani P., Saeidi A., Ram M.R., Velu V. (2017). CD8+ T cells of chronic HCV-infected patients express multiple negative immune checkpoints following stimulation with HCV peptides. Cell. Immunol..

[125] Saeidi A., Buggert M., Che K.F., Kong Y.Y., Velu V., Larsson M., Shankar E.M. (2015). Regulation of CD8+ T-cell cytotoxicity in HIV-1 infection. Cell. Immunol..

[126] Khan A., Jagannath C. (2017). Analysis of host-pathogen modulators of autophagy during *Mycobacterium tuberculosis* infection and therapeutic repercussions. Int. Rev. Immunol..

[127] Huang J., Brumell J.H. (2014). Bacteria-autophagy interplay: A battle for survival. Nat. Rev. Microbiol..

[128] Gutierrez M.G., Master S.S., Singh S.B., Taylor G.A., Colombo M.I., Deretic V. (2004). Autophagy is a defense mechanism inhibiting BCG and *Mycobacterium tuberculosis* survival in infected macrophages. Cell.

[129] Saito Y., Murata-Kamiya N., Hirayama T., Ohba Y., Hatakeyama M. (2010). Conversion of *Helicobacter pylori* CagA from senescence inducer to oncogenic driver through polarity-dependent regulation of p21. J. Exp. Med..

[130] Kalisperati P., Spanou E., Pateras I.S., Korkolopoulou P., Varvarigou A., Karavokyros I., Gorgoulis V.G., Vlachoyiannopoulos P.G., Sougioultzis S. (2017). Inflammation, DNA damage, *Helicobacter pylori* and gastric tumorigenesis. Front. Genet..

[131] Lewinska A., Wnuk M. (2017). *Helicobacter pylori*-induced premature senescence of extragastric cells may contribute to chronic skin diseases. Biogerontology.

[132] Terebiznik M.R., Raju D., Vázquez C.L., Torbricki K., Kulkarni R., Blanke S.R., Yoshimori T., Colombo M.I., Jones N.L. (2009). Effect of *Helicobacter pylori*’s vacuolating cytotoxin on the autophagy pathway in gastric epithelial cells. Autophagy.

[133] Galmiche A., Rassow J., Doye A., Cagnol S., Chambard J.C., Contamin S., de Thillot V., Just I., Ricci V., Solcia E. (2000). The N-terminal 34 kDa fragment of *Helicobacter pylori* vacuolating cytotoxin targets mitochondria and induces cytochrome c release. EMBO J..

[134] Raju D., Hussey S., Ang M., Terebiznik M.R., Sibony M., Galindo-Mata E., Gupta V., Blanke S.R., Delgado A., Romero-Gallo J. (2012). Vacuolating cytotoxin and variants in Atg16L1 that disrupt autophagy promote *Helicobacter pylori* infection in humans. Gastroenterology.

[135] Romano P.S., Cueto J.A., Casassa A.F., Vanrell M.C., Gottlieb R.A., Colombo M.I. (2012). Molecular and cellular mechanisms involved in the *Trypanosoma cruzi*/host cell interplay. IUBMB Life.

[136] Romano P.S., Arboit M.A., Vázquez C.L., Colombo M.I. (2009). The autophagic pathway is a key component in the lysosomal dependent entry of *Trypanosoma cruzi* into the host cell. Autophagy.

[137] Onizuka Y., Takahashi C., Uematsu A., Shinjo S., Seto E., Nakajima-Shimada J. (2017). Inhibition of autolysosome formation in host autophagy by *Trypanosoma cruzi* infection. Acta Trop..

[138] Andrade D.V., Gollob K.J., Dutra W.O. (2014). Acute Chagas Disease: New Global Challenges for an Old Neglected Disease. PLoS Negl. Trop. Dis..

[139] Sbaraglini M.L., Vanrell M.C., Bellera C.L., Benaim G., Carrillo C., Talevi A., Romano P.S. (2016). Neglected Tropical Protozoan Diseases: Drug Repositioning as a Rational Option. Curr. Top. Med. Chem..

[140] Albareda M.C., Olivera G.C., Laucella S.A., Alvarez M.G., Fernandez E.R., Lococo B., Viotti R., Tarleton R.L., Postan M. (2009). Chronic human infection with *Trypanosoma cruzi* drives CD4+ T cells to immune senescence. J. Immunol..

[141] Albareda M.C., Perez-Mazliah D., Natale M.A., Castro-Eiro M., Alvarez M.G., Viotti R., Bertocchi G., Lococo B., Tarleton R.L., Laucella S.A. (2015). Perturbed T Cell IL-7 Receptor Signaling in Chronic Chagas Disease. J. Immunol..

[142] Cardillo F., Falcao R.P., Rossi M.A., Mengel J. (1993). An age-related gamma-delta T cell suppressor activity correlates with the outcome of autoimmunity in experimental *Trypanosoma cruzi* infection. Eur. J. Immunol..

[143] González F.B., Calmon-Hamaty F., Nô Seara Cordeiro S., Fernández Bussy R., Spinelli S.V., D’Attilio L., Bottasso O., Savino W., Cotta-de-Almeida V., Villar S.R. (2016). *Trypanosoma cruzi* Experimental Infection Impacts on the Thymic Regulatory T Cell Compartment. PLoS Negl. Trop. Dis..

[144] Fülöp T., Larbi A., Pawelec G. (2013). Human T cell aging and the impact of persistent viral infections. Front. Immunol..

[145] Chidrawar S., Khan N., Wei W., McLarnon A., Smith N., Nayak L., Moss P. (2009). Cytomegalovirus-seropositivity has a profound influence on the magnitude of major lymphoid subsets within healthy individuals. Clin. Exp. Immunol..

[146] Karrer U., Mekker A., Wanke K., Tchang V., Haeberli L. (2009). Cytomegalovirus and immune senescence: Culprit or innocent bystander?. Exp. Gerontol..

[147] Weinberger B., Lazuardi L., Weiskirchner I., Keller M., Neuner C., Fischer K.H., Neuman B., Würzner R., Grubeck-Loebenstein B. (2007). Healthy Aging and Latent Infection with CMV Lead to Distinct Changes in CD8+ and CD4+ T-Cell Subsets in the Elderly. Hum. Immunol..

[148] Olsson J., Wikby A., Johansson B., Löfgren S., Nilsson B.O., Ferguson F.G. (2001). Age-related change in peripheral blood T-lymphocyte subpopulations and cytomegalovirus infection in the very old: The Swedish longitudinal OCTO immune study. Mech. Ageing Dev..

[149] Wikby A., Johansson B., Olsson J., Löfgren S., Nilsson B.O., Ferguson F. (2002). Expansions of peripheral blood CD8 T-lymphocyte subpopulations and an association with cytomegalovirus seropositivity in the elderly: The Swedish NONA immune study. Exp. Gerontol..

[150] Derhovanessian E., Theeten H., Hähnel K., Van Damme P., Cools N., Pawelec G. (2013). Cytomegalovirus-associated accumulation of late-differentiated CD4 T-cells correlates with poor humoral response to influenza vaccination. Vaccine.

[151] Frasca D., Diaz A., Romero M., Landin A.M., Blomberg B.B. (2015). Cytomegalovirus (CMV) seropositivity decreases B cell responses to the influenza vaccine. Vaccine.

[152] Trzonkowski P., Myśliwska J., Szmit E., Wiȩckiewicz J., Łukaszuk K., Brydak L.B., Machała M., Myśliwski A. (2003). Association between cytomegalovirus infection, enhanced proinflammatory response and low level of anti-hemagglutinins during the anti-influenza vaccination- An impact of immunosenescence. Vaccine.

[153] Furman D., Jojic V., Sharma S., Shen-Orr S.S., Angel C.J., Onengut-Gumuscu S., Kidd B.A., Maecker H.T., Concannon P., Dekker C.L. (2015). Cytomegalovirus infection enhances the immune response to influenza. Sci. Transl. Med..

[154] Wald A., Selke S., Magaret A., Boeckh M. (2013). Impact of human cytomegalovirus (CMV) infection on immune response to pandemic 2009 H1N1 influenza vaccine in healthy adults. J. Med. Virol..

[155] Redeker A., Remmerswaal E.B.M., van der Gracht E.T.I., Welten S.P.M., Höllt T., Koning F., Cicin-Sain L., Nikolich-Žugich J., Ten Berge I.J.M., van Lier R.A.W. (2017). The Contribution of Cytomegalovirus Infection to Immune Senescence Is Set by the Infectious Dose. Front. Immunol..

[156] Chaumorcel M., Lussignol M., Mouna L., Cavignac Y., Fahie K., Cotte-Laffitte J., Geballe A., Brune W., Beau I., Codogno P. (2012). The Human Cytomegalovirus Protein TRS1 Inhibits Autophagy via Its Interaction with Beclin 1. J. Virol..

[157] McFarlane S., Aitken J., Sutherland J.S., Nicholl M.J., Preston V.G., Preston C.M. (2011). Early induction of autophagy in human fibroblasts after infection with human cytomegalovirus or herpes simplex virus 1. J. Virol..

[158] Mouna L., Hernandez E., Bonte D., Brost R., Amazit L., Delgui L.R., Brune W., Geballe A.P., Beau I., Esclatine A. (2016). Analysis of the role of autophagy inhibition by two complementary human cytomegalovirus BECN1/Beclin 1-binding proteins. Autophagy.

[159] Levine B., Liu R., Dong X., Zhong Q. (2015). Beclin orthologs: Integrative hubs of cell signaling, membrane trafficking, and physiology. Trends Cell Biol..

[160] Kyei G.B., Dinkins C., Davis A.S., Roberts E., Singh S.B., Dong C., Wu L., Kominami E., Ueno T., Yamamoto A. (2009). Autophagy pathway intersects with HIV-1 biosynthesis and regulates viral yields in macrophages. J. Cell Biol..

[161] Gannagé M., Dormann D., Albrecht R., Dengjel J., Torossi T., Rämer P.C., Lee M., Strowig T., Arrey F., Conenello G. (2009). Matrix Protein 2 of Influenza A Virus Blocks Autophagosome Fusion with Lysosomes. Cell Host Microbe.

[162] Leib D.A., Gobeil P.A.M. (2012). Herpes simplex virus γ34.5 interferes with autophagosome maturation and antigen presentation in dendritic cells. MBio.

[163] Matsunaga K., Saitoh T., Tabata K., Omori H., Satoh T., Kurotori N., Maejima I., Shirahama-Noda K., Ichimura T., Isobe T. (2009). Two Beclin 1-binding proteins, Atg14L and Rubicon, reciprocally regulate autophagy at different stages. Nat. Cell Biol..

[164] Singh V., Finke-Isami J., Hopper-Chidlaw A.C., Schwerk P., Thompson A., Tedin K. (2017). *Salmonella* co-opts host cell chaperone-mediated autophagy for intracellular growth. J. Biol. Chem..

